# Chemo-immunoablation of solid tumors: A new concept in tumor ablation

**DOI:** 10.3389/fimmu.2022.1057535

**Published:** 2023-01-12

**Authors:** Liangliang Meng, Yingtian Wei, Yueyong Xiao

**Affiliations:** ^1^ Department of Radiology, the First Medical Center, Chinese PLA General Hospital, Beijing, China; ^2^ Department of Radiology, Chinese PAP Hospital of Beijing, Beijing, China

**Keywords:** chemo-immunoablation, tumor, immunotherapy, chemoablation, tumor microenvironment

## Abstract

Chemical ablation was designed to inject chemical agents directly into solid tumors to kill cells and is currently only used clinically for the palliative treatment of tumors. The application and combination of different drugs, from anhydrous ethanol, and glacial acetic acid to epi-amycin, have been clinically tested for a long time. The effectiveness is unsatisfactory due to chemical agents’ poor diffusion and concentration. Immunotherapy is considered a prospective oncologic therapeutic. Still, the clinical applications were limited by the low response rate of patients to immune drugs and the immune-related adverse effects caused by high doses. The advent of intratumoral immunotherapy has well addressed these issues. However, the efficacy of intratumoral immunotherapy alone is uncertain, as suggested by the results of preclinical and clinical studies. In this study, we will focus on the research of immunosuppressive tumor microenvironment with chemoablation and intratumoral immunotherapy, the synergistic effect between chemotherapeutic drugs and immunotherapy. We propose a new concept of intratumoral chemo-immunoablation. The concept opens a new perspective for tumor treatment from direct killing of tumor cells while, enhancing systemic anti-tumor immune response, and significantly reducing adverse effects of drugs.

## 1 Introduction

**Figure 1:**
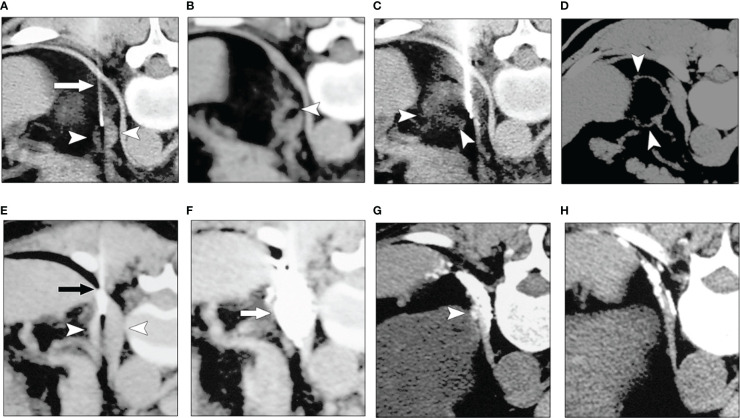
Chemical ablation of left adrenal adenoma with anhydrous alcohol **(A–D)**. **(A)** Transverse CT image obtained with the patient in a prone position showed a fine needle (arrow) in the left adrenal mass (arrowheads). **(B)**, CT image obtained after the first injection of alcohol revealed a focal collection of alcohol in the adrenal tumor (arrowhead). **(C)**, CT image obtained after the third ethanol injection shows the distribution of alcohol in tumor (arrowheads) was more diffuse than in **(B, D)** Follow-up CT image obtained one year after **(A–C)** revealed that most of the lesion (arrowheads) was replaced by fatty tissue. Chemical ablation for left adrenal metastases from the renal carcinoma after total left nephrectomy **(E–H)**. **(E)** A transverse CT scan with the patient in a prone position showed a needle (arrow) in the left adrenal mass (arrowheads). **(F)** The image obtained after the second injection of acetic acid and iodized oil showed good compound permeation (arrow). **(G)** Six months later, the follow-up CT image showed a remaining dotlike lesion (arrowhead), and iodized oil was within the diaphragm. **(H)** One year after A and B, a follow-up CT image showed the lesion had fully resolved (4). (Zhang JS et al, *AJR*, 2008).

Imaging-guided techniques have promoted the development of physical and chemical ablation-based therapies. Thermal ablation includes microwave ablation, radiofrequency ablation, laser ablation, cryoablation, and irreversible electroporation with voltage pulses (1–3). Chemical ablation refers to the procedure of inactivating tumor cells by injecting chemical ablative agents directly into the solid tumor using a fine needle under imaging guidance. Early chemical ablative agents used anhydrous alcohol or glacial acetic acid, which can directly cause protein coagulation and necrosis of tumor cells when injected into the tumor. They were commonly used to treat small tumors such as the liver and adrenal adenoma (**Figure 1**) (4, 5). Because the fluidity of ablative agents such as anhydrous alcohol in the tumor is difficult to control, the intra-tumor drug retention time which is short and unevenly distributed. The efficacy in larger tumors were not satisfactory. Hence, the intra-tumor injection of chemotherapy drugs alone is challenging to achieve the effect of radical treatment and is more often used for palliative treatment, which may limit the application of this technology.

Tumor immunotherapy alters the state and function of the immune system by inhibiting or stimulating specific pathways or molecules of the immune system, which in turn enhances its ability to clear tumor cells. Bacillus Calmette-guérin (BCG) was the first immunotherapy drug approved by the FDA for superficial bladder cancer (6). Other emerging immunotherapeutic approaches include tumor vaccines, immune adjuvants, lytic virus therapy, various cytokines, and Chimeric Antigen Receptor T-Cell Immunotherapy (CAR-T) (7). In particular, the recent development and clinical application of cytotoxic T lymphocyte-associated antigen-4 (CTLA-4) and programmed cell death protein-1/ligand-1 (PD-1/PD-L1) immune checkpoint inhibitors once brought a new era of tumor treatment. However, the lower overall response rate, the obstruction of the blood-brain barrier, and the frequent occurrence of adverse reactions to systemic therapy have significantly limited their wide application (8). The presence of intratumoral immunotherapy has solved these problems by using imaging-guided puncture technology to inject immune agents percutaneously into the tumor directly with a fine needle of 22G-25G to increase the drug concentration in the tumor area instantly or by locally injecting immune adjuvant into the tumor to permanently change or reverse the immune microenvironment of the tumor and improve the effect of systemic immunotherapy (9). Intratumoral immunotherapy can minimize the adverse impact on the whole body (10). Major types of intratumoral immunotherapy include pattern recognition receptor agonists, Toll-like receptor agonists, interferon gene stimulating factor agonists, retinoic acid-induced gene receptor agonists, immunomodulatory antibodies, oncolytic viruses, messenger ribonucleic acids (mRNA and DNA), immune cells, etc.

A radical cure is difficult to achieve with the single application of chemical ablation. Tumor cell death is significantly associated with the tumor microenvironment (TME). The action of TME is a fundamental cause of immune tolerance and escape of tumor cells. Theoretically, immunotherapy may be more effective by altering the TME. Numerous previous studies have also confirmed the synergistic antitumor effects of chemotherapy and immunotherapy (11, 12). In this review, we summarize the various approaches and applications of local chemoablation and intratumoral immunotherapy. We innovatively propose a new concept of chemo-immunoablation. The combination of local chemoablation and intratumoral immunotherapy under the guidance of imaging to permanently improve the TME will produce powerful local and systemic anti-tumor effects. The new concept provides a new perspective and solution for oncology treatment.

## 2 The profile of the TME

In the process of tumor development, the interaction between the immune system and the tumor is usually subdivided into three stages: immune clearance, immune homeostasis, and immune escape (13). In the period of immune clearance, cancerous cells will be monitored and attacked by the immune system and eliminated through innate immunity and adaptive immunity; the tumor cells surviving immune clearance reach a balance with the immune system, i.e., enter immune homeostasis; tumor escape occurs when the tumor cells gain the preponderance in the struggle with the immune cells. These processes are closely related to the TME tumor cells inhabit, which can also be understood as the soil in which tumor cells live. The “seed and soil” hypothesis proposed by Stephen Paget in 1889 has provided the basis for the concept of the TME (14–16). The TME consists of numerous immune cells, mesenchymal cells, extracellular matrix, active media, and tumor cells. It can be roughly divided into an immune microenvironment dominated by immune cells and a non-immune microenvironment dominated by fibroblasts. The specific TME can promote tumor proliferation and metastasis by affecting the proliferation of tumor cells, regulating the expression level of metastasis-related genes, inducing angiogenesis, and promoting the degradation of the extracellular matrix (17).

Tumor cells are infiltrated by immune cells and secrete inflammatory mediators, forming an inflammatory microenvironment. The immune cell components are complex and diverse, including T lymphocytes (70%-80%) and B lymphocytes (10%-20%), macrophages (5%-10%), natural killer (NK) cells (<5%), and dendritic cells (DCs), and Myeloid Derived Suppressor Cells (MDSC). Lymphocytes that infiltrate locally in the tumor are usually called tumor infiltration lymphocytes (TILs) (18). Regulatory T cells (Tregs) and tumor-associated macrophages (TAMs) mediate tumor immunosuppression and promote tumor proliferation (19, 20). Tregs are a subpopulation of CD4+ T lymphocytes that constitutively express interleukin 2 (IL-2) receptor (CD25), CTLA-4, and Foxp3, with two primary functions: immune incompetence and immunosuppression (20). TAMs are essential in mediating the onset of tumor inflammation and progression (19). Ample evidence supports a key role for MDSCs in immune suppression in cancer, as well as their prominent role in tumor angiogenesis, drug resistance, and promotion of tumor metastases (21, 22).

In addition to inflammatory and immune cells, stromal cells such as fibroblasts, vascular smooth muscle cells, and endothelial cells constitute the non-immune microenvironment of tumors and provide protection and support for tumor development. Cancer-associated fibroblasts (CAFs) are the most abundant host cells in the microenvironment. Distinct from normal fibroblasts, CAFs are characterized by the expression of α-smooth muscle actin (α-SMA) and fibroblast-activating protein (FAP), which secrete a large number of growth factors such as VEGF and TGF-β and mediate ECM remodeling (23). Endothelial cells are the basis of tumor angiogenesis. The TME can modify the expression of endothelial cell genes to favor angiogenesis, including inhibiting the expression of genes such as plexin and pro-fibronectin 1 (24). VEGF induces vascular leakage by binding to VEGF receptors on endothelial cells and promotes the proliferation and migration of tumor vascular endothelial cells (25).

In addition, numerous studies have found that TME is closely related to tumor immunotherapy, and immunosuppressive TME may lead to the failure of tumor immunotherapy (26, 27). Inadequate oxygen supply, glucose depletion, mammary gland accumulation, and extracellular vesicles due to tumor cell metabolism can lead to nutrient deficiency and dysfunction of tumor-infiltrating lymphocytes, leading to immunosuppression (26). TME with a highly enriched in CD8^+^ T cells plus high levels of PD‐L1 expression are more likely to respond to inhibition of PD-1/PD-L1 (28, 29). In addition, different modulation strategies can convert the immunodominant TME into an immunostimulatory TME and promote the effects of systemic immunotherapy. For example, local TLR7/8 agonists can enhance the recruitment and activation of immune cells in tumors and polarize anti-tumor immunity to Th1 response, augmenting the effect of immune checkpoint inhibitors (30). In addition, local chemical ablation can significantly change the TME, eliminate and inhibit the growth of tumor cells and destroy their survival conditions, including the death of fibroblasts, etc.

## 3 Local physical and chemical ablation of solid tumors

Thermal-based physical therapies are the most commonly used methods for tumor ablation and are divided into thermal ablation and cryoablation according to the ablation temperature. As an interventional technique, thermal ablation has been widely used to treat tumors in various body organs and has achieved good results. The commonly used thermal ablation methods include radiofrequency ablation, microwave ablation, laser ablation, high intensity focused ultrasound (HIFU). The ablation tissue necrosis is characterized as coagulative necrosis. Radiofrequency ablation is the earliest and most widely used thermal ablation treatment (31). Its basic principle is to introduce a high-frequency oscillating current into tumor tissues through ablation electrodes so that local tissue ions and polarized molecules oscillate rapidly with the alternating direction of the current, resulting in tissue frictional heat generation; cells die immediately when the temperature exceeds 70°C, and the cell membrane dissolves, and intercellular water evaporates when the temperature reaches 100°C, resulting in tissue disintegration and charring. Microwave ablation has high efficiency, short action time, and complete tumor necrosis, one of the most common thermal ablation methods. Laser ablation is the coagulation and necrosis of tumor cells by emitting/scattering laser light through optical fibers and converting it into heat energy in the lesion (32).

Early cryoablation devices used liquid nitrogen as the refrigerant, requiring thicker probes (not less than 3 mm in diameter). They were used primarily for intraoperative cryotherapy under direct vision of tumors but were limited in their application because of the inability to determine the ablation boundary. Recent cryotherapy devices are represented by the argon-helium knife, which is based on the Joule-Thomson effect and uses high-pressure argon gas at room temperature to rapidly produce temperatures as low as -185°C at the tip of the needle and up to 50°C at the tip of the needle by rewarming with high-pressure helium gas, i.e., accelerating tumor necrosis through the cycle of freezing-rewarming (33). The principle of cryoablation is the formation of ice crystals within the cell interstitium, destruction of organelles, rupture of cell membranes, and tissue necrosis. The release of tumor antigens due to cryoablation stimulates the body’s immune response and is part of today’s tumor immunology research. Cryoablation can be guided by ultrasound, CT, or MRI to clearly show the size of the ice ball and its relationship with the tumor tissue. A multi-needle combination of cryoablation can realize conformal tumor ablation (34). Cryoablation is currently a method that can adequately cover large tumor volumes and is painless and well-tolerated by patients. Cryoablation is easier to control than thermal ablation in treating soft tissue and superficial tumors.

Although cold or heat-based thermal ablation techniques have been widely used in clinical practice, their development is limited by the non-selective physical destruction of local tissues, the most common shortcoming of which is that in addition to the inactivation of tumor tissues within the ablation area, adjacent normal tissue structures such as blood vessels, nerves, intestinal ducts, pancreaticobiliary ducts, etc. are damaged, especially in the lesions of essential structures such as the hilum, hilar region, and retroperitoneum. Physical ablation may lead to severe complications such as bleeding, and the risks of ablation far outweigh the benefits.

Irreversible electroporation (IRE) is an ambient temperature physical ablation technique that is performed by applying high voltage electrical pulses to tumor cells through electrodes to produce nano-scale irreversible electroporation on the cell membrane, resulting in the death of tumor cells. Its most significant advantage is that it causes minimal damage to major anatomical structures in the ablation area, such as arteries, veins, nerves, bile ducts, trachea, intestines, and ureters, and can effectively protect the structural integrity of the vasculature (3). IRE is particularly suitable for the ablation of tumors located in essential structures such as the pancreas, hilar region, and retroperitoneum, which cannot be treated by other physical ablations (35). There are some significant problems in the clinical application of IRE: IRE can induce action potentials, leading to periodic violent muscle contractions and even inducing seizures, causing changes in the position of the ablation needle, affecting the ablation effect and possibly damaging the surrounding tissue.

In addition to some critical cellular components of TME that play a crucial role in tumor drug resistance, the extracellular matrix (ECM) plays a vital role in tumor growth, migration, and drug resistance. The ECM’s collagenous tissue, hyaluronic acid, and proteoglycan structures constitute a natural “barrier” for tumor cell self-protection, preventing anti-tumor drugs from reaching tumor cells. In addition, the dense tumor cells and the lack of an intact lymphatic reflux system result in high interstitial fluid pressure (IFP) between tumor cells, increasing the resistance of drugs from the blood vessels to the interstitial tissue space. Therefore, it is difficult for the anti-tumor drugs in the blood circulation to reach the tumor tissues.

**Figure 2:**
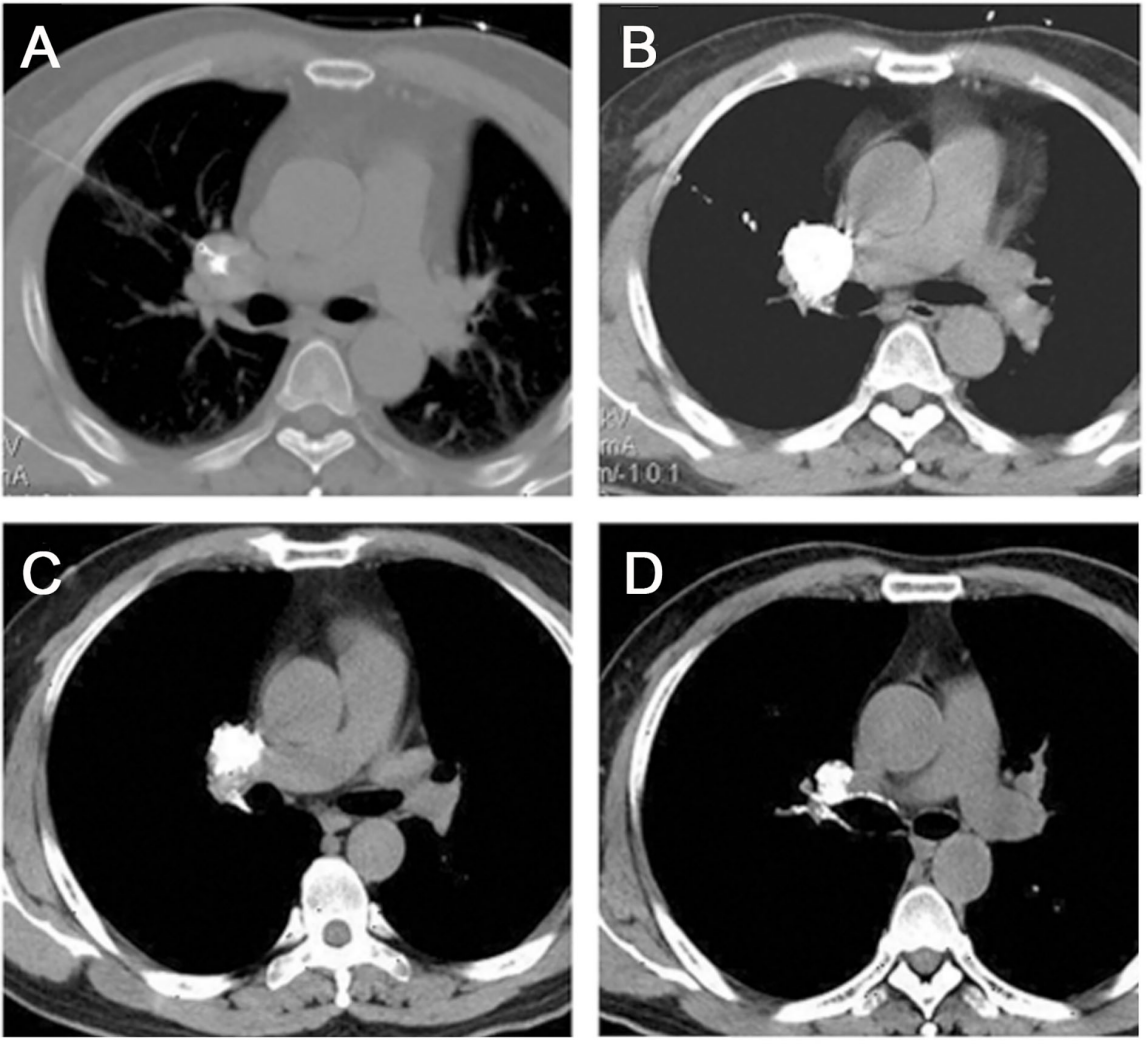
Chemical ablation of a lymphatic node metastasis in the right pulmonary hilar region. **(A)** Transverse CT before chemical ablation showed a tumor at the lung hilum. The needle tip was located in the center of the target LN. **(B)** The image obtained after a single injection of the ethanol-ethiodol-drugs mixture showed good LN permeation. **(C-D)** At 6 and 12 months after ablation, the tumor size gradually decreased. (Liu SR et al., Technol Cancer Res Treat, 2013).

Intratumoral chemical ablation is a method of *in situ* inactivations of the tumor by percutaneous puncture of tumor tissue under imaging guidance and direct injection of ablative agents into the tumor internally (4, 5, 36) (**Figure 2**). This technique overcomes the higher IFP of tumor tissue. It can directly inject highly concentrated drugs into the tumor at high pressure, fully contacting tumor cells and improving the local anti-tumor effect of medications. It is suitable for primary and metastatic tumors in different body parts (37, 38). The most commonly used ablative agents were tumor cytotoxic agents and protein coagulants in the early stage. For example, Liu et al. used a mixture of cytotoxic drugs and chemical agents to treat metastatic lymph nodes and achieved a tumor CR of 91.7% after 12 months (36). These early chemoablation treatments were only limited to the direct killing of tumor cells and not considering the impact on the TME. This limits the application of intratumoral chemical ablation. Intratumoral chemical ablation therapy may be an option for patients unsuitable for surgical resection and local physical ablation treatment. Transcatheter arterial chemoembolization (TACE) can be performed first for blood-rich tumors to embolize the tumor vessels and then additional chemical ablation for the residual lesions, leading to complete necrosis. Combining chemoablation with TACE is a palliative treatment with limited efficacy for larger tumors.

## 4 Application of intratumoral immunotherapy

Although radiotherapy and chemotherapy are seen as first-line treatment options for unresectable tumors, the success of immunotherapy, especially immune checkpoint inhibitors (ICIs), has gradually changed the game. Still, not all patients received benefits from ICIs therapy. However, intratumoral immunotherapy is now available in many preclinical and clinical trials and is gradually demonstrating its advantages (39–43). Although most immunotherapy is currently administered intravenously, the larger dosage of agents delivered intravenously increases the incidence of adverse events systemically and may even lead to interruption of the treatment. Intratumoral immunotherapy, however, may avoid this issue. Like intratumoral chemoablation, intratumoral immunotherapy generally utilizes a 20 or 22 G percutaneous needle to inject the immune agent directly into the tumor under ultrasound, CT, or MRI guidance.

In addition to targeting tumor cells, tumor immunotherapy may also affect normal tissues in various body organs, leading to autoimmune toxicity, including rash, pneumonia, colitis, pituitary inflammation, and hepatitis (44). In addition, some agents can cause varying degrees of cardiotoxicity, including fatigue, myalgia, chest pain, dyspnea, and syncope (45). Compared to intravenous administration, the smaller dosage of intratumoral immunologic agents brings less toxicity and adverse effects.

The mechanism of immunotherapy consists of three phases. First is the recruitment phase of DCs, where cytokines, Toll-like receptor (TLR)-9 agonists, and STING agonists will induce local IFN-γ release, leading to the recruitment and activation of DCs in the tumor area. This is followed by an initiation phase where these DCs will activate T cells, which are allowed to activate when anti-CTLA-4 antibodies block T cells and dendritic cell suppressor signals. Finally, PD-1/PD-L1 antibodies will inhibit inhibitory signals from tumor cells, blocking the immune escape of tumor cells and exposing them to cytotoxic T-cell attacks (46).

The basic principle of cytokine therapy for tumors is to use cytokines to stimulate the body’s immune system to kill tumor cells. The two main types of cytokines with immune-activating activity are interleukin (IL) and interferon (IFN), which is more commonly used in clinical practice. For example, IL2 can activate and increase T cells and NK cells. It was first used in metastatic kidney cancer and melanoma (47). On the other hand, Intratumoral cytokine therapy has been gradually applied to the clinic in recent years and has achieved good efficacy. For example, an ongoing phase III clinical trial on intratumoral IL-2 injection for melanoma (NCT03233828) has achieved excellent results.

The main biological functions of pattern recognition receptors (PRR) include activation of complement, phagocytosis, initiation of cell activation and inflammatory signaling, and induction of apoptosis. TLR agonists are the most studied intratumoral immunotherapeutic tools, including TLR3, TLR5, and TLR9 agonists. Stimulation of TLR9, for example, triggers the production of pro-inflammatory cytokines (e.g., IFN-I, IL6, IL12, TNF-a), thereby activating innate immune factors, including DC and NK cells. In turn, antigen cross-presentation by mature DCs initiates the immune system and ultimately kills tumor cells (48). In a preclinical trial, Carmine Carbone et al. found that local application of TLR agonists significantly changed the immunosuppressive TME in pancreatic cancer. The therapy produced a robust synergistic anti-tumor immune response at both *in situ* and distant tumor sites through combination with systemic PD-1 inhibitors (49). Antoni Ribas et al. demonstrated that SD101, a TLR9 agonist, significantly improved the response to systemic Pembrolizumab immunotherapy in patients with advanced melanoma. By local injection, extensive immune activation was induced in the TME, and a durable anti-tumor response in distant lesions was produced (9).

Oncolytic viruses are transgenic or naturally occurring viruses that can preferentially infect tumor cells, thereby causing tumor cell lysis without affecting normal cells, and are genetically engineered to express immunomodulatory proteins to promote immune system activation. The first FDA/European Medicines Agency (EMA) approved intra-tumor immunotherapy was T-VEC, a herpes-derived lysing virus that expresses human GM-CSF. It showed objective responses and overall survival benefits in stage IIIb-IVM1a melanoma (50, 51). Other lytic viruses, such as Pexa-Vec, a poxvirus-derived OV that also encodes human GM-CSF, have shown their ability to respond significantly in hepatocellular carcinoma (52). T-VEC is a genetically modified herpes simplex virus type 1 intratumoral lysing virus-based therapy that has been extensively tested in preclinical and clinical trials with promising results (53). In patients with unresectable metastatic melanoma, local intratumoral injections of T-VEC show superior durable response rates to subcutaneous GM-CSF (10).

Immune checkpoint inhibitors bind to immune checkpoints on T cells or tumor cells to block tumor escape from immune system and restore the body’s anti-tumor immune response. Currently, antibodies or inhibitors against PD-1, PD-L1, CTLA-4, T cell immunoglobulin, and mucin domain-containing protein 3 (TIM3) are commonly used. Still, the lower response rate determines that only a few patients will benefit (54, 55). Immune checkpoint inhibitors have revolutionized the landscape of tumor immunotherapy, and the anti-tumor effects of intratumoral applications, in particular, have been demonstrated in numerous preclinical trials. Recently, there have been many clinical trials focusing on the clinical application of intratumoral immune checkpoint inhibitors with encouraging preliminary results, including the phase I clinical trial of cemiplimab, an intratumoral anti-PD-1 agent (NCT03889912), and the phase I clinical trial of Nivolumab, an intratumoral anti-PD-1 agent (NCT03316274), Phase II and Phase III clinical trials of the intratumoral anti-PD-1 drug ipilimumab or Nivolumab in combination with the anti-CTLA-4 drug Ipilimumab (NCT04090775, NCT03755739), anti-CD40 inhibitor Selicrelumab in combination with the anti-PD-L1 inhibitor Atezolizumab (NCT03892525) and other (56). For patients with allogeneic transplants, intratumoral anti-PD-1 treatment combined with TLR9 agonist can ensure a strong anti-tumor immune response while avoiding severe transplant rejection caused by systemic immunotherapy, reflecting the unique effectiveness and safety of intratumoral injection (57). In contrast, the immunosuppressive TME may be responsible for the low systemic immune response and immune checkpoint inhibitor therapy failure. Preclinical studies have shown that resistance to systemic immune checkpoint blockade therapy can be broken through local delivery of immunostimulatory products such as oncolytic viruses, cytokines, and PRR agonists (58, 59).

For advanced cancer, including giant tumors, metastases, or recurrent tumors, we usually perform another biopsy at an appropriate site to confirm the pathological results. We recommend genetic testing to identify whether the patient is suitable for immunotherapy or targeted therapy. For these patients, chemical ablation or intratumoral alone can hardly achieve eradication and is only a palliative treatment. In contrast, a better therapeutic effect can be achieved if combined with immunotherapy, especially since intratumoral immunotherapy can ensure sufficient drug concentration and low systemic toxicity in the tumor region. The lower adverse effects of intratumoral immunotherapy also provide an opportunity to combine intratumoral with systemic immunotherapy to provide better treatment options for multiple metastatic or recurrent lesions in the body.

## 5 Intratumoral ablative agents and immunotherapy drugs

### 5.1 Preparation of chemotherapy drugs

**Figure 3:**
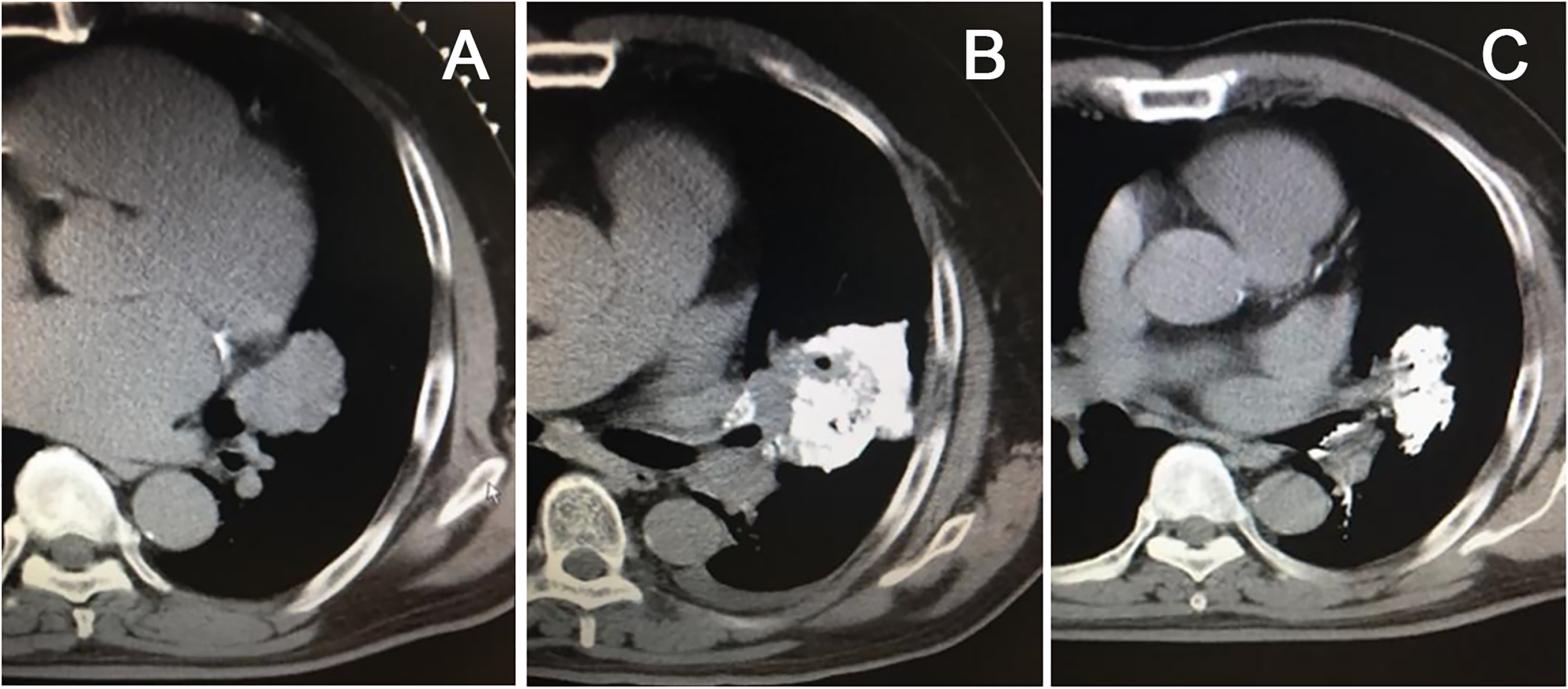
Chemical ablation of left-sided central lung cancer, CT scan showed a large lesion in the hilar region **(A)**, chemical ablation was performed with cisplatin plus epiamphetamine, and iodinated oil was used for tracing **(B)**. The mass shrank significantly three months after treatment **(C)**.

Early tumor cytotoxic drugs for chemical ablation were prepared by mixing chemotherapeutic drugs according to tumor cytology type with a small amount of iodinated oil, which can be injected into tumor or metastatic lymph nodes percutaneously to kill tumor cells by slow release of anti-tumor drugs in tumor tissues and reduce the toxic damage to patient’s body (**Figure 3**). Liu et al. used a percutaneously punctured injection of chemical ablation drugs to treat pulmonary metastatic lymph nodes. They injected a mixture of 99.9% absolute alcohol (5ml), Doxorubicin (10mg), and Lipiodol ultra-fluid (2ml) into metastatic lymph nodes using a 21G or 22G Chiba needle. The injection dose was based on the tumor size and should gradually fill up the tumor under the surveillance of CT (36). The disadvantages were the precise amount of drug in the tumor, the release time was not easy to grasp, the diffusion area of the ablative agent was not controllable, and repeated injections were often required. The mechanism of chemotherapy is based on the blockage of the synthesis of DNA, RNA, and proteins, which are the basic components of chromatin in cells, and the subsequent prevention of the division and proliferation of cancer cells, thus achieving the therapeutic goal. The most commonly used chemotherapeutic drugs include alkylating agents, antimetabolites, anti-tumor antibiotics, methylating drugs, platinum compounds, etc. The most widely used medications for local intratumoral injection include platinum compounds such as cisplatin and oxaliplatin and the anti-tumor antibiotic Epiamphenicoln, etc. (37, 60, 61). In our institution, for larger tumors such as central lung cancer and hepatocellular carcinoma in the hilar region, we perform puncture biopsies in advance when allowed, remove the tumor tissue and then perform cytology culture and drug sensitivity tests, which in turn will determine and screen the most effective chemotherapeutic agent before chemical ablation is performed. A promising approach is using polymer gels as drug carriers to prolong intra-tumor drug residence time in an early double-blind trial of 20 patients treated with a mixture of fluorouracil and polymer gel given intratumorally for basal cell carcinoma. They compared the efficacy of two doses of fluorouracil (0.5ml vs. 0.25ml) and found that patients in the 0.5ml dose group achieved 80% histological cure (62). This gel treatment with local chemotherapeutic agents has lower chemotherapeutic toxicity compared to systemic chemotherapy. In clinical practice, 20-26G needles are commonly used for puncture and injection of chemotherapeutic agents, and the dose administered for local therapy is much lower than the dose routinely administered intravenously (about 5-20 mg of cisplatin intratumorally, compared to 160-220 mg typically administered intravenously).

Commonly used protein coagulants include anhydrous ethanol, glacial acetic acid, etc. The mechanism is to cause coagulation of tumor cells, cytoplasmic dehydration, and tumor cell necrosis (63). Anhydrous ethanol is easy to diffuse and retain for small tumors due to its relatively homogeneous internal structure and often pseudo-envelope, resulting in complete tumor necrosis. In contrast, for larger tumors, the diffusion of the ablative agent is limited due to the mixed composition and fibrous segregation within the tumor. Ohnishi et al. showed that the effect of 50% glacial acetic acid on tumor cells was three times that of anhydrous ethanol (63). Since glacial acetic acid is more permeable than anhydrous ethanol, it can easily penetrate the envelope to the surrounding normal tissues and cause severe pain when treating small lesions. The tracer property of glacial acetic acid is relatively poor. Still, dilution with an equal amount of water-soluble contrast agent can improve the tracer property of glacial acetic acid. CT scans can monitor the dispersion of high-density ablative agents in the tumor, whether injected into blood vessels or extra-tumor tissues. Therefore, anhydrous ethanol should be used as an ablative agent for tumors less than 3 cm in diameter. Glacial acetic acid can be used as an ablative agent for tumors larger than 3 cm in diameter. In addition, some Chinese scholars used a dilute hydrochloric acid compound to destroy cancer cells, which was five times more effective than 50% glacial acetic acid and 15 times more effective than anhydrous ethanol in coagulating cancer tissue proteins, and its efficacy is significantly better than anhydrous ethanol and glacial acetic acid. We have observed in clinical practice that the ablative formulation should achieve good tracer properties and slow drug release and injection rate to ensure adequate diffusion of the ablative agent in tumor tissues. **Table 1** shows some clinical trials of chemical ablation of tumors at different sites, with the commonly used chemical ablation reagents and ablation protocols and doses (36, 64–71).

**Table 1:** Selected clinical trials of chemical ablation of different tumors.

Authors	Tumor type	Agents	Dosage	Clinical outcome	Population	Locations
Liu SR, Xiao YY, Le Pivert PJ, et al	Metastatic lymph node carcinoma	Ethanol-ethiodol-doxorubicin emulsion	The actual injected dose is based on the intraprocedural CT imaging with a maximum allowed dose of 10 ml per session	Complete response rates of 91.7% were obtained at 12 months after therapy	36	Beijing, China
Xinglu Xu, Xiuling Li, Xin Ye, et al	Recurrent mediastinal nodal metastasis	Solution (9:1) of ethiodol	An injection– validation–injection method with 1 mL initially injected using 22G PTC needle	Chemoablation as salvage treatment after post-radiotherapy relapse is efficacious and safe	31	Shandong, China
Lonny Yarmus, Christopher Mallow, Jason Akulian, et al	Malignant Airway Obstruction Secondary to Non-small Cell Lung Cancer	Paclitaxel	An average of 3.4 injections given for a total dose of 1.5 mg of paclitaxel	Significantly less stenosis postprocedure	20	Maryland, United States, North Carolina, United States
John F Thompson, Peter Hersey, Eric Wachter, et al	Metastatic melanoma	Rose Bengal	10% w/v Rose Bengal in saline at a dose of 0.5 ml/cc	Response rate was dose dependent,	11	Camperdown, New South Wales, Australia
Sergio Cavalheiro, Concezzio Di Rocco, Sergio Valenzuela, et al	Craniopharyngiomas	Interferon-alpha	The number of cycles varied from 1 to 9, and the total dose applied per cycle was 36 MU	Clinical and radiological improvement was achieved in 76% of the cases	60	São Paulo, Brazil
D F Ierardi, M J S Fernandes, I R Silva, et al	Cystic craniopharyngiomas	Interferon-alpha	A 3 MU of IFN-α was injected and was repeated in alternating days for 12 times as a cycle.	Complete reduction of tumor size in 11 patients The concentration of sFasL was increased in patients with the tumor size reduction	21	São Paulo, Brazil
Hiroshi Takahashi, Fumio Yamaguchi, Akira Teramoto, et al	Craniopharyngioma in children	Bleomycin	Bleomycin was administered 2 weeks postoperatively via the Ommaya reservoir at a dose of 5 mg every other day until total dose of 40 mg	The children with excellent clinical outcomes have no recurrence during follow-up from 21 to 26 years	7	Kanagawa, Japan
Christian Duvillard, Philippe Romanet, Alain Cosmidis, et al	Locally recurrent head and neck tumors	Intratumoral cisplatin and epinephrine	Cisplatin (1 mg/mL) Epinephrine (0.02 mg/mL) (1 mL/cm3 of tumor; maximum volume, 50 mL)	Eight objective responses were registered among the 14 patients	14	Dijon, France.
Spyridoy Voulgaris, Melpomeni Partheni, Michalis Karamouzis, et al	Malignant brain gliomas	Doxorubicin	Doxorubicin 0.5 mg was administered every 24 hours on days 1 to 10	Objective radiologic response was observed in 50% patients	10	Rion, Greece.
K Engelmann, M G Mack, R Straub , et al	Malignant liver tumors	Cisplatin/epinephrine injectable gel	Liver metastases (mean volume of 42 ml with a mean of 5.1 injections) HCC nodules (mean volume of 22.1 ml with a mean of 3.25 treatments)	Substantially higher local therapy control rate for HCC compared to colorectal metastases.	16	Frankfurt, Germany
M T Farrés, T de Baere, C Lagrange, et al	Primary and secondary liver tumors	Mitoxantrone	10-20 mg of mitoxantrone mixed with 0.5 ml of contrast medium	No complications, no change in 11 cases	15	Villejuif, France

As mentioned above, chemotherapy is still the main drug used in clinical practice. Its treatment principle is limited to the cytotoxicity of the drug itself, which is often ineffective for larger or poorly diffused tumors. With the studies and recognition of the TME, more and more researchers focus on changing the immunosuppressive TME. By injecting drugs into local tumors, not only can we directly kill tumor cells, but another significant achievement is to improve theimmune activity in the TME, promote the body’s anti-tumor immune response, and enhance the cytotoxic T lymphocytes to kill tumors.

### 5.2 Preparation of intratumoral immunotherapy drugs

Intratumoral immunotherapy creates high blood concentrations locally in the lesion, which can improve the efficacy of immunotherapy, reduce immune-related adverse effects, and expand the population benefiting from immunotherapy, especially by facilitating the use of dose-limiting drugs. Types of intratumoral immunotherapeutic agents include oncolytic viruses, anti-CTLA-4 antibodies, anti-CD40 agonist antibodies, immunostimulatory TLRs and STING agonists, immunostimulatory nanoparticles, oncolytic peptides and viruses, mRNAs encoding cytokines (72).

In a randomized phase II study in 30 patients with unresectable primary HCC, the doses of Pexa-Vec were 10^8^ and 10^9^ plaque-forming units per milliliter, respectively. Both doses had a good safety profile, with the most common adverse event being flu-like symptoms. The higher dose of Pexa-Vec resulted in a 62% intrahepatic response rate compared to the low-dose group and was significantly associated with improved overall survival (73). In another phase I clinical trial (NCT04612530), intratumoral injection of the TLR9 agonist IMO-2125 in combination with IRE for pancreatic cancer, the operator administered 8 mg of IMO-2125 intratumorally to each patient one week before IRE was performed (74).

Immune checkpoint inhibitors typically target PD-1/PD-L1 as well as CTLA-4. Systemic monotherapy or other approaches, such as radiotherapy, have been widely used in the clinic, but the intravenous approach can lead to highly frequently occurring systemic immune-related adverse reactions. Recent randomized phase III trials of ipilimumab injections of 3 mg/kg versus 10 mg/kg did show that higher doses of systemic anti-CTLA-4 were associated with higher immunotoxicity (75). The dose of intratumoral CTLA-4 inhibitor (50 μg) is only 1/8 of that of intravenous injection with the same efficacy, which significantly reduces systemic immune-related adverse events, and CTLA-4 inhibitor is usually mixed with mineral oil adjuvant Montanide ISA-51 VG and shaken into an emulsion before injection (76). Intratumoral injections of CTLA-4 inhibitors have been clinically effective to date, with demonstrated efficacy and safety in phase I clinical trials for the treatment of advanced melanoma, and also showed an enhanced systemic anti-tumor immune response induced by local injections (77). Specifically, the highest intra-tumoral tolerated dose of the CTLA-4 inhibitor ipilimumab and IL-2 was assessed based on toxicity over the first three weeks. Finally, the tolerated dose of intratumoral Ipilimumab was 2 mg. A 2 mg dose of IL-2 was injected into the same lesion three times per week for two weeks and then twice weekly for six weeks (77). In the phase II clinical trial of cryoablation combined with intratumoral immunotherapy for metastatic prostatic adenocarcinoma (NCT04090775), PD-1 inhibitor monoclonal antibody nivolumab and anti-CTLA-4 monoclonal antibody ipilimumab were sequentially injected directly into the tumor immediately following local cryoablation. The injection regimen for Nivolumab was 10 mg/mL for 1 ml, and for Ipilimumab was 5 mg/mL for 1 ml. This way, dendritic cells can initiate a cell-mediated systemic immune response in combination with cytotoxic killer T-cells. In **Table 2**, we detailed the officially registered clinical trials on the intratumoral injection of immune checkpoint inhibitors. We listed the tumor types, drug injection protocols, doses, and efficacy evaluation criteria of different trials (77–81).

**Table 2:** Registered clinical trial of intratumoral injection of immune checkpoint inhibitors (ICIs).

Clinical Trial Number	Status	Tumor type	Agents	Dosage of intratumoral ICIs	Outcome Measures	Population	Locations
NCT04090775	Completed	Prostatic Adenocarcinoma	Drug: Opdivo Drug: Yervoy Drug: cytoxan	Nivolumab Injectable 10mg/mL, only 1 mL injected. Ipilimumab: Injectable 5mg/mL, only 1 mL injected.	Primary endpoint: PSA decline Efficacy: iRECIST criteria	12	California, United States Michigan, United States
NCT03982121	Withdrawn	Colorectal Cancer Metastatic	FOLFOX regimen Nivolumab Ipilimumab GLA-SE	Ipilimumab 5 or 10 or 25 mg	MTD Recommanded phase 2 dose (RP2D)		Val De Marne, France
NCT03892525	Terminated	Recurrent B-Cell Non-Hodgkin Lymphoma	Selicrelumab Atezolizumab	Escalated dose in intratumoral injection, every 3 weeks, for 3 cycles	Optimal dose of intratumoral Selicrelumabin Doses of Atezolizumab IV PFS/OS/SAE	4	Créteil, France …..
NCT03889912	Recruiting	Cutaneous Squamous Cell Carcinoma Basal Cell Carcinoma	Cemiplimab		NCI CTCAE v5 Selection of the recommended dose of cemiplimab	61	Phoenix, Arizona, United States
NCT03755739	Recruiting	Hepatocarcinoma Lung Cancer Melanoma Renal Cancer Head and Neck Cancer Pancreas Cancer Ovarian Cancer Colo-rectal Cancer Cervical Cancer Breast Cancer	ICIs such as Pembrolizumab	150mg via intra-tumor fine needle injection in 5 min, every 3 weeks.	OS CR rate PFS Duration of remission Disease control rate	200	Guanzhou, Guangdong, China
NCT03707808	Completed	Solid Tumor Metastases to Soft Tissue	Intratumoral injection of autologous CD1c (BDCA-1)+ myDC	Intratumoral injection of ipilimumab and avelumab	ORR of intratumoral injected CD1c (BDCA-1) + myDC, avelumab, and ipilimumab plus iv nivolumab	9	UZ Brussel, Jette, Brabant, Belgium
NCT03316274	Completed	Kaposi Sarcoma HIV/AIDS	Intra-lesional injection of nivolumab	10mg in 1 mL injection into a KS lesion in the skin, every 2 weeks	Changes in PD-1 expression as determined by immunohistochemistry in lesions	12	San Francisco, California, United States
NCT03233152	Recruiting	Glioblastoma	Ipilimumab	Yervoy, 50 mg/10 mL solution Opdivo, 40 mg/4mL solution	PFS/OS	6	Brussel, Brussels, Belgium
NCT03058289	Active	Breast Cancer Head and Neck Cancer Squamous Cell Carcinoma Lymphoma Pancreatic Cancer Liver Cancer Colon Cancer Lung Cancer Bile Duct Cancer Chordoma of Sacrum Sarcoma	INT230-6 anti- PD-1 antibody anti- CTLA-4 antibody	The anti-PD-1 antibody will be added concomitantly with INT230-6 as noted in cohort DEC and DEC2	CTCAE v.4.03) (Scale 1 to 5) Preliminary Efficacy: Control or Regression of Injected Tumors by Measurement of Length, Width and Height Determine pharmacokinetic parameter	110	California, United States Maryland, United States Massachusetts, United States …..
NCT02977156	Completed	Metastatic Tumor Advanced Tumor	Pexa-Vec Ipilimumab	Four dose levels of ipilimumab will be tested in dose escalation step: 2.5mg, 5mg, 7.5mg, 10mg, 20mg or 40 mg	Dose Limiting Toxicities ORR/PFS/TTP/OS	22	Bordeaux, France Lyon, France Paris, France Pierre-Bénite, France Villejuif, France
NCT02890368	Terminated	Solid Tumors Mycosis Fungoides Melanoma Merkel-cell Carcinoma Squamous Cell Carcinoma Breast Carcinoma Human Papillomavirus- Related Malignant Neoplasm Soft Tissue Sarcoma	TTI-621 Monotherapy TTI-621+ PD-1/PD-L1 Inhibitor TTI-621 + pegylated interferon-#2a TTI-621 + T-Vec TTI-621 + radiation	TTI-621 will be given in combination with PD-1/PD-L1 Inhibitor	Optimal TTI-621 delivery regimen Frequency and severity of adverse events Preliminary evidence of anti-tumor activity of TTI-621	56	California, United States New York, United States
NCT02857569	Active	Stage III/IV Melanoma	Ipilimumab IT Ipilimumab IV Nivolumab IV	0.3mg/kg IT Ipilimumab injection every 3 weeks	6-months treatment-related grade 3-4 toxicity EFS.	90	Gustave Roussy, Villejuif, Val De Marne, France
NCT01672450	Completed	Melanoma	Intratumoral Ipilimumab and Interleukin-2	Ipilimumab (0.1, 0.25, 0.5, 1, 2, mg) IT weekly x 8 weeks	Starting and Ending measurements of treated lesions Starting and ending measurement of untreated lesions	12	Salt Lake City, Utah, United States
NTR6119	Active	Cervical cancer	Durvalumab	Intratumourally injected 5, 10 and 20 mg	MTD DLTs		Amsterdam, the Netherlands.

progression free survival, PFS, overall survival, OS, serious adverse event, SAE, Objective response rate, ORR, Time To progression, TTP, event-free survival, EFS, Maximum tolerated dose, MTD, Complete response, CR, Dose-limiting toxicities, DLTs.

PD-1/PD-L1 inhibitors are currently the most focused immunotherapies and have achieved brilliant results in various tumor types (8, 82). Intratumoral injections can likewise reduce systemic immune adverse effects while ensuring better efficacy. There are fewer reports of intratumoral injections of PD-1/PD-L1 inhibitors, but all have achieved encouraging results. Intratumoral injections were administered in the same way and at the same dosage. Lambros Tselikas et al. reported the therapeutic effect of intratumoral immuno-injections for various types of tumors, such as melanoma and lung cancer. They performed intratumoral immunotherapy on 100 patients. The number of injections and the dosage used varied for different tumors. The needle used for injections was generally 22 G. Preoperatively, 1% lidocaine was used for local anesthesia, and simultaneous biopsy was performed if possible (72).

### 5.3 Application of controlled release drug delivery systems

Local delivery of immunotherapeutic drugs requires consideration of the needle’s thickness, the tumor’s size and nature, and the drug release kinetics. Biocompatible polymers are ideal delivery pathways for immunotherapeutic drugs. Suitable delivery vehicles can be constructed for intra-tumor immunoadjuvants, including particle suspensions, polymer-drug couplings, and amphiphilic block polymers that self-assemble into nanoparticles (46). Early injectable formulations were mainly based on degradable and non-degradable particulate suspensions. Functional groups on polymeric backbones (e.g., polycarboxylic acid polymers) or terminal groups at the nano-scale can prepare drug-polymer couplings, thereby reducing systemic toxicity and increasing target accumulation. Hydrogels are another long-established and continuously innovative polymerization technology. Hydrogels are soft materials with extremely high water content and a cross-linked polymer backbone structure that allows for structural integrity while allowing for controlled degradation, stimulus responsiveness, biocompatibility, and controlled drug release (83). The structure of an ideal hydrogel system should allow injectability in 18G or more delicate puncture needles, supervised regulated release for at least a week or so, binding to specific immune adjuvants, and a regulated release time of about a week. Yu et al. applied a P(Me-D-1MT)-PEG-P(Me-D-1MT) hydrogel system that delivered an anti-PD-1 antibody and IDO inhibitor intratumorally. It maintained a longer controlled release time and better drug concentration, enhanced anti-tumor response, and inhibited tumor growth (84).

There is also the issue of visualization of controlled release systems such as hydrogels under imaging guidance. Currently, fluorescence is used to observe drug diffusion and controlled release in many animal experiments, but it does not apply to humans with *in-vivo* tumors (85). A mixture of iodinated oil and poly(lactic-co-glycolic acid) nanoparticles (PEEP) was used for the intratumoral injection of anti-CTLA-4 antibodies, with an average droplet size of approximately 42 +/-5 μm. Clear visualization by iodized oil under CT or X-ray enables precise observation of the diffusion range and extent of the drug (86). Iodide oil radiopaque concentrations are typically in the range of 100 mg/mL. They are applied at doses similar to local chemical ablation, but the presence of a contrast agent may affect the controlled release kinetics of immune pharmaceuticals. For combination into polymers, most iodides have hydroxyl groups that can be used for chemical conjugation or spatial binding using hydrophobic/hydrophilic properties to form polymeric conjugates (87). For intratumoral injections, a non-permanent contrast agent is recommended, as permanent contrast agents may affect the assessment of future efficacy.

## 6 The concept of chemo-immunoablation

**Figure 4:**
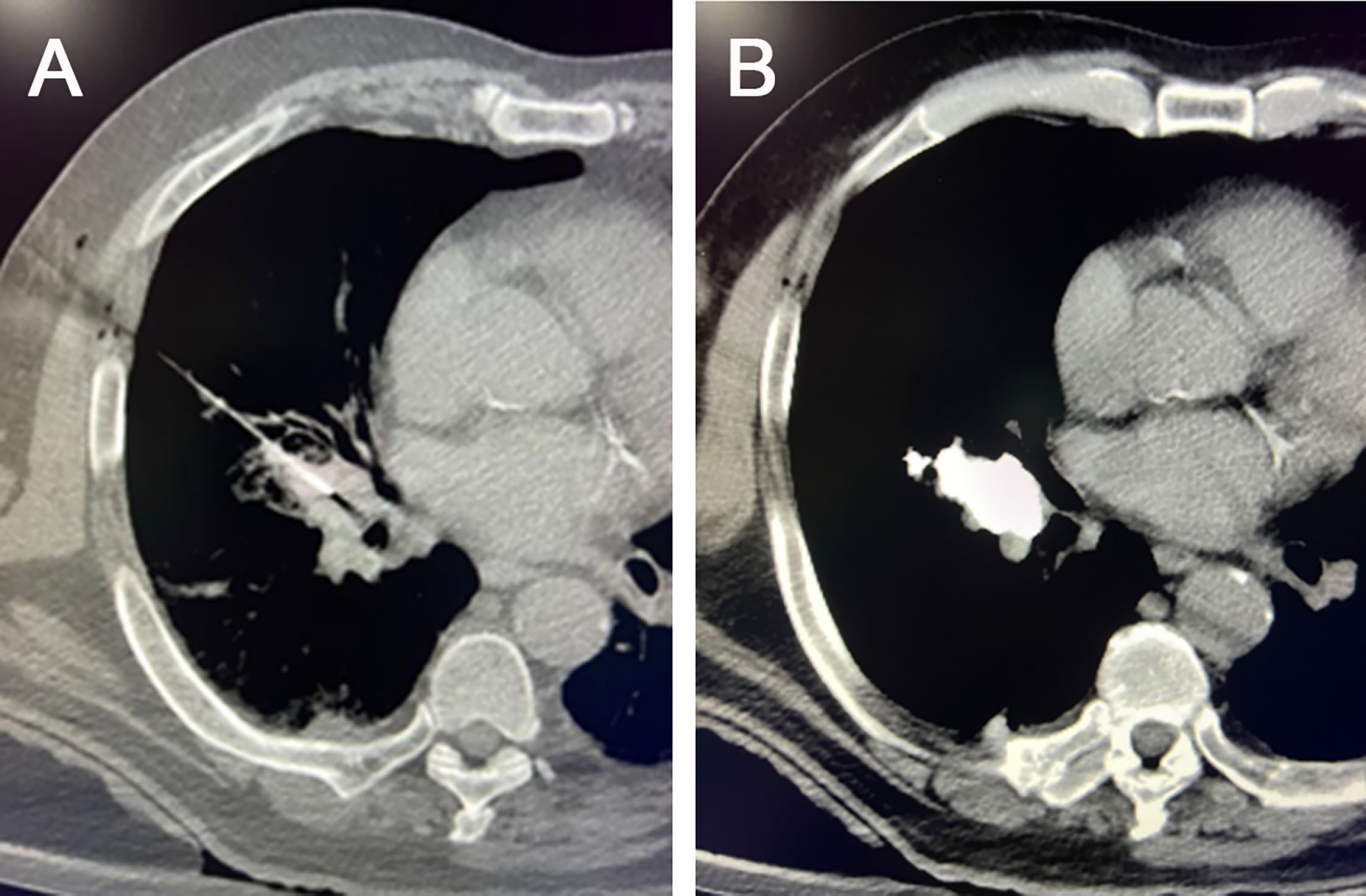
Chemo-immunoablation of right-sided central lung cancer. **(A)** A 22G puncture needle was used to reach the main part of the tumor, **(B)** followed by chemical ablation with cisplatin plus epoetin and intratumoral immunotherapy with 5-10 mg of pembrolizumab *via* needle sheath. A small amount of iodine oil was used as a tracer to observe the diffusion of the drugs.

Systemic chemotherapy or immunotherapy has proven its excellent contribution in inhibiting tumor growth, modifying the local TME, and prolonging patient survival (17, 88). Potential synergistic antitumor effects have been demonstrated between chemotherapy and immunotherapy (89). However, a large proportion of systemic chemotherapy or immunotherapy regimens are causing adverse events. In addition, the immunosuppressive TME hinders the response to immunotherapy in different patients. With the mature application of local chemoablation for solid tumors and the research and development of intratumoral immunotherapy in improving TME, it is possiple to reverse immunosuppression to enhance systemic immune response. The concept of chemo-immunoablation was born. The proposed chemo-immunoablation improved chemo-ablation or intratumoral immunotherapy alone by changing the immunosuppressive TME. Chemo-immunoablation refers to the combination of chemo-ablation and immunotherapy to improve the local TME and enhance the systemic anti-tumor immune response by injecting chemo-ablative agents into larger tumors or tumors near important organ structures. By injecting immunosuppressive drugs and immune adjuvants locally, it is possible to inactivate the tumor *in situ* while playing a systemic anti-tumor immune response, under the premise that physical ablation is not possible or suitable (**Figure 4**). The effect of chemotherapy is to improve the local TME and enhance the systemic anti-tumor immune response. Compared with intratumoral chemotherapy or intratumoral immunotherapy alone, chemo-immunoablation in one procedure will have several significant advantages.

(1) Chemical ablation combined with immunotherapy can significantly avoid immunosuppressive TME. Applying immune adjuvants or immunosuppressive agents can stimulate the systemic anti-tumor immune response, which can have a systemic anti-tumor effect through local treatment. Using local immune adjuvant can avoid the systemic immunosuppressive state and create opportunities for combined systemic immunotherapy.
(2) The diffusion advantage of intratumoral drug injection over intravenous injection. Intratumoral injection of chemotherapeutic agents or immune drugs enables rapid diffusion into the tumor tissue, allowing total exposure of the drug to the tumor cells. They were combined with a drug-controlled release system such as hydrogel, which can provide the ideal sustained drug release time for tumor treatment.
(3) Advantages of drug concentration. Compared with intravenous drug delivery, intratumoral local drug delivery has some advantages in terms of drug concentration: Firstly, intratumoral drug delivery is the direct contact between chemotherapy or immunotherapy drugs and targeted tumor cells, which can significantly reduce the dose of medications used compared with intravenous injection while ensuring the local drug concentration; secondly, the reduction of total drug dose also reduces the medical cost of patients and provides more opportunities for combining other drugs. Moreover, smaller drug concentrations can significantly reduce complications such as inflammation and autoimmune diseases.
(4) The advantages of injection with synchronized biopsy to detect intratumoral immune status and individualized targeted therapy. Another unique benefit of intratumoral chemo-immunoablation is that synchronized biopsy can be performed during drug administration, which can clarify the gene expression of tumor tissues and the state of the immune microenvironment, providing a new theoretical basis for the improvement of individual chemo-immunoablation protocols. By performing drug sensitivity tests on the biopsied tumor tissue, the optimal chemotherapy protocol can be adjusted for the patient. In addition, applying a coaxial needle puncture biopsy significantly reduced the possibility of tumor needle tract metastasis.

The success of intratumoral chemo-immunoablation requires, in addition to the type of drug, the determination of the sequence, dose and frequency of administration in clinical work. We should also develop individualized treatment protocols for multiple tumors or different types of tumors.There are still relatively few studies related to chemo-immunoablation, but the few that have been done have been highly successful. XL Yu et al. loaded docetaxel (a chemo-agent) and cynomorium songaricum polysaccharide (CSP, an immunomodulator) into zein nanoparticles coated by a green tea polyphenols/iron coordination complex (GTP/FeIII, a photothermal agent). The therapy eliminated the primary tumor, prevented tumor recurrence and significantly inhibited tumor metastasis (90). This chemo-immunoablation could promote the release of damage-associated molecular patterns (DAMPs). CRT, ATP, and HMGB1 are released by the dying tumor cells. And the CSP could assist the DAMPs in inducing the maturation of DCs and facilitate the intratumoral TILs to clear up the residual or disseminated tumor cells (90).

The success of chemo-immunoablation also faces some technical challenges. Firstly, the surgeon must have solid, stable, and superior puncture skills. The needle should follow the principle of a stepwise approach, and the number of needle revisions should be minimized during the arrival of the needle into the tumor to avoid puncturing vital vascular organs and reduce surgical complications and failure rates. Secondly, the target tumor should be of sufficient size. Generally, it should be larger than 1 cm so that the puncture needle can enter the center of the tumor and inject into it. The tumor is too tiny for puncture or injection failure to occur easily. In addition, the preparation of ablation drugs and the dosage of injected drugs should be flexible according to the tumor size and the diffusion of drugs. Usually, when we perform chemical immunoablation, we mix iodinated oil evenly with the drug and then puncture and administer the medication under the guidance of CT. The distribution and actual diffusion of the drug can be observed in real-time through the high-density contrast of iodinated oil. If necessary, the direction of the needle will be adjusted, and the drug will be administered again to ensure that the drug is in complete contact with the entire tumor as much as possible. In addition, chemo-immunoablation for intracranial CNS lesions is rarely carried out at present, mainly due to the deep location of the tumor, the obstruction of the skull, and the possible irreversible damage to the nervous system during the procedure.

## 7 Summary and prospects

The concept of intratumoral chemo-immunoablation has provided new prospects for treating some particular sites and types of tumors. According to pathology, genetic testing, and drug sensitivity experiments, imaging-guided percutaneous chemo-immunoablation can, on the one hand, kill tumor cells to the maximum extent through direct contact with a high concentration of sensitive chemotherapeutic drugs. On the other hand, it can reverse the immunosuppressive TME, enhance the body’s anti-tumor immune response and kill residual tumors through intratumoral immunity. There is a robust synergistic anti-tumor effect between chemotherapeutic and immunotherapeutic agents. Its major advantage is that local high-concentration administration avoids systemic chemotherapy or immune-related adverse reactions, bringing new possibilities and options for patients who cannot undergo systemic chemotherapy or immunotherapy. The fewer complications and lower medical costs also open up new opportunities for more combinations of drugs. As preclinical trials are refined, and more clinical trials are conducted, it is hoped that chemo-immunoablation will become a unique new technology that can be flexibly applied to clinical tumor treatment.

## Author contributions

LM conceived and designed the study. LM drafted the entire manuscript. YW is responsible for Figures and Image processing. YW and YX reviewed and revised the manuscript. All authors contributed to the article and approved the submitted version.
